# Socioeconomic status modifies the association between adherence to the Mediterranean diet and cognitive outcomes: results from the Collaborative PROMED-COG Pooled Cohorts Study

**DOI:** 10.1007/s00127-025-02993-2

**Published:** 2025-09-29

**Authors:** Federica Prinelli, Marianna Noale, Silvia Conti, Adele Ravelli, Giuseppe Sergi, Stefania Maggi, Chiara Ceolin, Lorraine Brennan, Lisette CPGM de Groot, Claire T. McEvoy, Caterina Trevisan

**Affiliations:** 1 Institute of Biomedical Technologies, National Research Council (CNR), Via Fratelli Cervi 93, 20054 Segrate, MI Italy; 2 Neuroscience Institute, Aging Branch, National Research Council (CNR), Viale Giuseppe Colombo 3, 35121 Padua, Italy; 3 Department of Medical Sciences, University of Ferrara, via Fossato di Mortara, 64/B - 44121 Ferrara, Italy; 4 Geriatric Unit, Department of Medicine, University of Padova (UNIPD), Via Giustiniani 2, 35128 Padua, Italy; 5 School of Agriculture and Food Science, Institute of Food and Health and Conway Institute, University College Dublin, Belfield, Dublin 4, Ireland; 6 Division of Human Nutrition, Wageningen University, Droevendaalsesteeg 4, 6708 PB Wageningen, Netherlands; 7 Centre for Public Health, Queen’s University Belfast, Belfast, Northern Ireland BT7 1NN UK; 8 The Global Brain Institute, Trinity College Dublin, Ireland & University of California, 1651 4th St, CA 94158, San Francisco, USA

**Keywords:** Mediterranean diet, Physical activity, Cognitive decline, Dementia incidence, Socioeconomic status, Retrospective harmonization, Pooled cohorts

## Abstract

**Background:**

This study examines whether adherence to the Mediterranean diet (MD), alone and combined with physical activity (MedEx), is associated with cognitive decline and dementia incidence, with socioeconomic status (SES) as a potential modifier.

**Methods:**

We included 8,568 subjects (mean age 72.3 ± 9.6 years, 52.4% female) from three pooled Italian population-based studies. MD adherence was assessed using the Panagiotakos algorithm. We analyzed the association of MD and MedEx adherence, both continuously and categorized in tertiles, with cognitive decline and incident dementia using Cox regression. SES modification was examined through interaction analysis and SES-stratified models.

**Results:**

Cognitive decline occurred in 38.1% of participants but was not associated with MD adherence. In SES-stratified analysis, among high SES individuals, each 2-point increase in MD adherence reduced cognitive decline risk by 14%, and high MD adherence was associated with a 48% reduction (HR 0.52, 95%CI 0.31–0.90). In this group, medium MedEx adherence reduced cognitive decline risk by 77% (HR 0.23, 95%CI 0.07–0.83). No significant association was found between MD/MedEx adherence and incident dementia (4.2%), regardless of SES.

**Discussion:**

SES may modify the relationship between MD and cognitive decline, with greater benefits observed in higher SES groups. Further studies, particularly in vulnerable populations, are needed to inform tailored preventive strategies for cognitive decline.

**Supplementary Information:**

The online version contains supplementary material available at 10.1007/s00127-025-02993-2.

## Background

In 2020, more than 55 million people worldwide were affected by dementia, and this number is expected to rise as life expectancy increases. Globally, cognitive decline and dementia are major global health problems and a leading cause of disability in older people. Thus, identifying modifiable risk factors and the most vulnerable population is crucial for developing effective prevention programs, particularly targeting individuals who would benefit most from health promotion interventions [1].

Currently known modifiable risk conditions for cognitive decline and dementia include vascular and metabolic factors, unhealthy behaviors, and low socioeconomic status (SES) [2]. Previous meta-analyses have shown that a healthy lifestyle, including a healthy diet such as the Mediterranean diet (MD) and regular physical activity (PA), is associated with a reduced risk of cognitive decline and dementia [3–6]. Low SES is also linked to higher incidence of dementia and cognitive impairment [7, 8]. It is characterized by chronic financial stress, limited access to nutritious foods, and systemic inequities that force cost-saving choices over quality foods. Additionally, the effects of SES on cognitive dysfunction may be also partly related to lifestyle differences. Indeed, socioeconomically disadvantaged people may be more likely to be engaged in risky behaviors—such as smoking, physical inactivity [9], and unhealthy diets [10, 11], compared with those who are more advantaged. Moreover, low SES may affect cognitive function by reducing the cognitive reserve, which is associated with lower levels of education or occupational complexity. This reduced cognitive reserve may delay the clinical manifestation of dementia-related brain changes [12, 13].

Some studies indicate that SES may influence the effect of lifestyle on health outcomes—such as cardiovascular disease and mortality—suggesting that the impact of healthy behaviors on cognition could vary by SES [14–16]. However, to our knowledge, very few studies have examined the role of SES in modifying the association between healthy behaviors, particularly diet, and neurocognitive outcomes [16–20]. The existing findings are heterogeneous, likely due to differences in cognitive outcomes measurements or social contexts. Addressing this gap is crucial for developing targeted dementia prevention programs that flatten SES-related inequalities while promoting healthy lifestyles.

In this study, we estimated the longitudinal associations between adherence to the MD—alone and in combination with PA—with cognitive decline and incident dementia. We also assessed whether SES modifies these associations, using data from a large sample of Italian adults and older people included in the PROtein enriched MEDiterranean diet to combat undernutrition and promote healthy neuroCOGnitive ageing in older adults (PROMED-COG) Pooled Cohort Study [21].

## Materials and methods

### Study population

The analytical sample was derived from three population-based longitudinal studies previously described [22, 23] and briefly outlined below. The Italian Longitudinal Study of Ageing (ILSA, [24]) is a population-based longitudinal study involving a random sample of 5,632 individuals aged 65–84 years in 1992–93 (baseline) identified from the population registers of eight municipalities in northern, central and southern Italy. Follow-up examinations were carried out in 1995–96 and 2000–01. The Progetto Veneto Anziani (Pro.V.A, [25], is a longitudinal study based on an age- and sex-stratified random sample of 3,099 adults aged ≥ 65 years living in northern Italy. The baseline assessment was carried out in 1995–97, with follow-up assessments in 1999–2000 and 2002–04. A passive follow-up until 2018 using regional health registers to derive hospitalization and mortality data was recently carried out. The Italian Bollate Eye Study [26]—Follow-up (BEST-FU; [27]), is a longitudinal study of 1,604 dementia-free community-dwelling individuals aged 40–74 years from the Lombardy region of northern Italy. The baseline assessment was performed in 1992–93, and the sample was followed up until 2012 using the electronic health records.

**Fig. 1:**
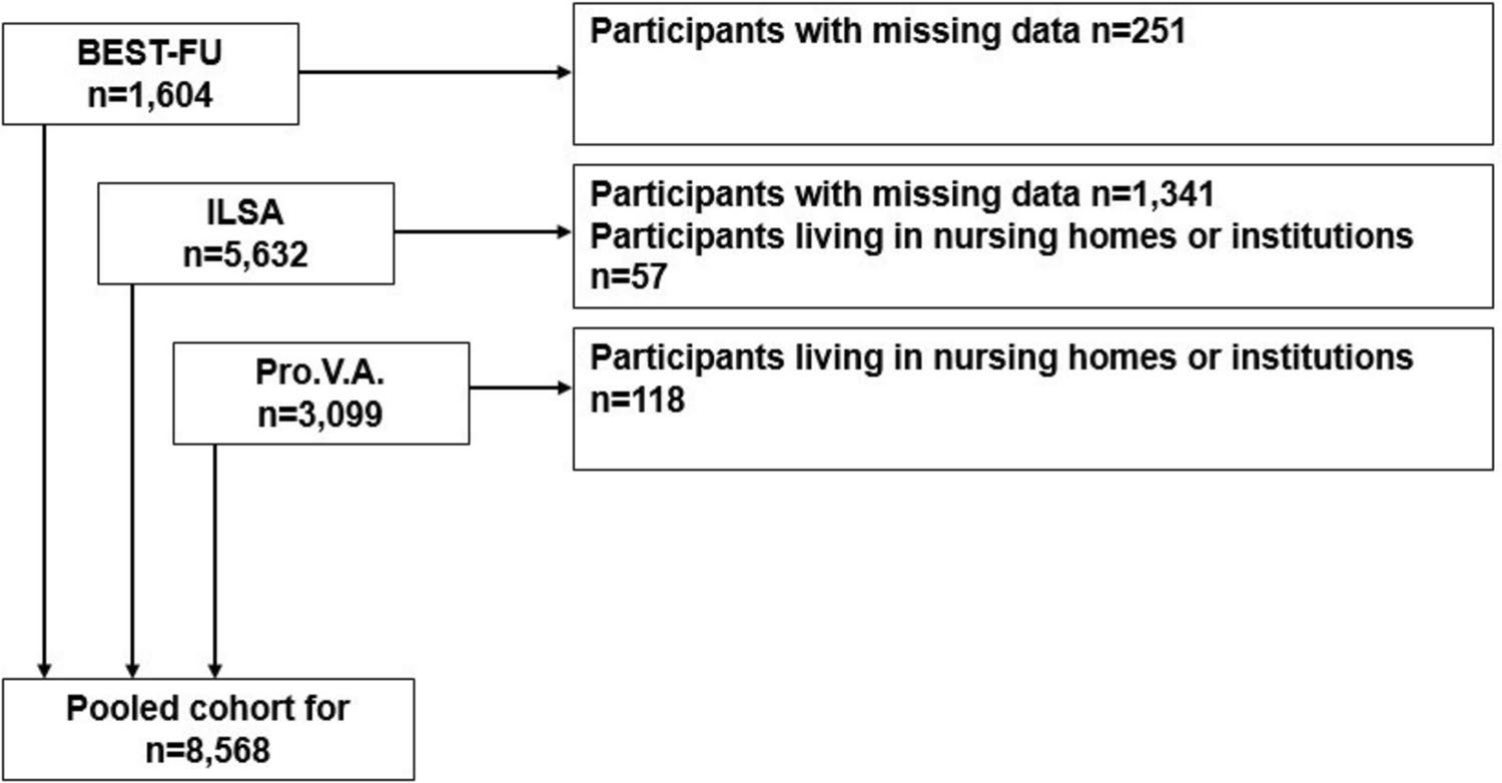
Selection of the final analytical sample

While the Pro.V.A. and ILSA studies included participants with dementia at baseline, the BEST-FU study only enrolled community-dwelling individuals who were free of dementia at baseline. However, all three studies recorded incident dementia during follow-up assessments. The characteristics of the three cohorts are shown in Table 1. For the purposes of the present study, we excluded individuals living in nursing homes (n = 118 enrolled in the Pro.V.A. study, and n = 57 in the ILSA study) and those with missing data on the main exposure and outcome variables (n = 1,341, from the ILSA study and n = 251, from the BEST-FU study), resulting in a final pooled sample of 8,568 community-dwelling individuals. Missing values for the ILSA study were due to the fact that some scales were administered during the clinical evaluation (Q3) of the screening assessment, after the personal interview (Q1) and the nurse visit (Q2) [24]; as some subjects withdrew from the study between Q1 and Q3, their Mini-mental state examination (MMSE) was not available and their assessment of dementia could not be performed. Data for the BEST-FU was missing due to the unavailability of dietary data (Fig. 1).

**Table 1:** Characteristics of the studies included in the PROMED-COG Pooled Cohorts Study, Italy, 1992–2023

Study	Study site	Baseline recruitment	Study population	Study design	Dietary assessment methods					Outcomes ascertainment
					Type	No. of items	Time frame	Components measured	Administration	
Italian Bollate Eye Study Follow up—BEST-FU [26]	Lombardy region, North of Italy	1992–1993	1693 age range 40–74 50.3% women	Prospective, passive follow-up (median 18 years)	FFQ	158	Past year	Frequency and portion size	Face-to-face interview	Dementia incidence (electronic health records)
Progetto Veneto Anziani—Pro.V.A. [25]	Veneto region, North of Italy	1995–1997	3099 age range 65–101 59.1% women	Prospective, active and passive follow-up (median 7 years)	FFQ	52	Past week	Frequency and portion size	Face-to-face interview	MMSE (clinical evaluation); dementia (electronic health records)
Italian Longitudinal Study of Ageing—ILSA [24]	North, Central and South of Italy	1992–1993	5632 age range 65–85 48.6% women	Prospective, active follow-up (median 8 years)	SFFQ	49	Past week	Frequency of standard portion size	Face-to-face interview	MMSE; dementia (clinical evaluation), clinical diagnosis of dementia

FFQ: food frequency questionnaire; SFFQ: semi-quantitative food questionnaire; MMSE: Mini-Mental State Examination

### Retrospective harmonization procedure

Retrospective data harmonization was performed according to the guidelines of Fortier et al. [28]. Seven sections of interest were defined and included as previously reported [22]: (i) general information (participant’s identification code, baseline date, follow-up dates); (ii) socio-demographic characteristics (sex, date of birth, education, main occupation, marital status). A harmonized socioeconomic status (SES) variable was defined, taking into account both education (primary school or middle school: score 1; high school or university or higher: score 2) and main occupation (blue collar worker or housewife: score 1; white collar worker: score 2) [29, 30]; a composite SES measure with a total score of 1,2 corresponded to SES = 1 (low); a total score of 3 corresponded to SES = 2 (medium); and a total score of 4 corresponded to SES = 3 (high); (iii) nutritional status (weight and height at baseline were used to calculate body mass index (BMI) using the standard formula (weight/height^2^); (iv) health status variables (hypertension, diabetes, hyperlipidemia, cardiovascular diseases (angina, ischemic heart disease, arrhythmia, peripheral artery disease, stroke), bowel or stomach diseases, liver or gallbladder diseases, chronic bronchitis or emphysema or asthma, cancer, bone or joint diseases, heart failure, depressive symptoms, limited mobility; the number of medications); (v) dietary habits (daily energy intake (kcal), daily consumption of foods and non-alcoholic beverages, food groups); (vi) lifestyle (smoking habits, alcohol consumption, physical activity); (vii) neurocognitive outcomes (cognitive performance (MMSE) [31] at baseline and follow-up; diagnosis of dementia at baseline and follow-up).

### Dietary data assessment and harmonization of data processing

The collection of dietary data in the three cohorts has been described in detail previously [23] and is briefly reported here. In the ILSA study, dietary data were collected by a trained interviewer using a 49-item food frequency questionnaire (FFQ). Participants reported how often they had eaten each food on average during the previous week. Alcohol consumption was measured in milliliters consumed per day. Food consumption was measured in standard portion sizes. In the Pro.V.A., a trained interviewer administered a 52-item FFQ, asking participants to report how often they had eaten each food on average in the previous week. The amount of food consumed was assessed using the standard portion size. Only for the Pro.V.A. study, where some dietary components had more than 20% missing values, medians by sex and age were imputed from the ILSA study (comparable to the Pro.V.A. study in terms of baseline year and study design). In the BEST-FU study, dietary habits in the year before recruitment were assessed by a trained interviewer using a FFQ (adapted from Willett’s questionnaire in the Nurses’ Health Study [32]) administered, consisting of a list of 158 food items. Participants were asked to report the average number of times they had consumed a given food item in the past year, using a seven-point frequency scale ranging from never to 4–5 times per day. The amount of food consumed was assessed by selecting a picture of a food portion. Details regarding the dietary data gathering in the three cohorts are reported in Table 1.

As described in our previous methodological paper [23], a step-by-step procedure was followed to obtain harmonized dietary data across the three datasets. To calculate the Panagiotakos MD score [33], foods were aggregated into eleven higher-order food groups (cereals, fruits, vegetables, potatoes, legumes, olive oil, fish, red meat, poultry, full-fat dairy products, and alcohol). In contrast to the original algorithm developed by Panagiotakos, which only included unrefined cereals, we included all types of cereals, as original variables for unrefined grains were not available for all studies. The individual foods and common food groups included in the calculation of the Panagiotakos score are shown in Supplementary Table S1. For the components considered to be typical of the MD (cereals, fruits, vegetables, potatoes, legumes, fish, olive oil), scores from 0 to 5 were assigned based on the monthly frequency of consumption (servings/month; 0 = no consumption, 1 = 1–4 servings/month, 2 = 5–8 servings/month, 3 = 9–12 servings/month, 4 = 13–18 servings/month, and 5 =  > 18 servings/month); for olive oil score = 0 was assigned in case of no consumption, 1 for rare consumption, 2 for < 1 times/week, 3 for 1–3 times/week, 4 for 4–5 times/week and 5 for daily use. For components considered to be far from the MD paradigm (red meat, poultry, full-fat dairy products), scores were assigned on an inverted scale (0 =  > 18 servings/month, 1 = 13–18 servings/month, 2 = 9–12 servings/month, 3 = 5–8 servings/month, 4 = 1–4 servings/month, and 5 = no consumption). For alcohol, a score was assigned based on daily consumption in ml (0 =  < 700 or 0 ml/day, 1 = 600 ml/day, 2 = 500 ml/day, 3 = 400 ml/day, 4 = 300 ml/day, and 5 < 300 ml/day). The overall MD score defined by Panagiotakos ranged from 0 to 55 and categorized according to the tertiles of its distribution (1: first tertile, low adherence; 2: second tertile, medium adherence; 3: third tertile, high adherence). Despite methodological heterogeneity in dietary data collection, FFQ structure and population characteristics, to ensure comparability across the three cohorts, tertiles of adherence were calculated within each individual study. This approach enabled relative classification of adherence to the Mediterranean diet to be based on internal distribution of scores, thereby minimising potential misclassification due to differences in dietary assessment instruments and enhancing the validity of pooled analyses.

A combination of MD adherence and physical activity behavior (MedEx) was also defined. The MD summary score [1–3] was summed with the dichotomized physical activity score (0 = less than 4 hours/week of physical activity; 1 = 4 or more hours/week of physical activity). The MedEx score thus ranged from 0 (low adherence to the MD and less than 4 hours/week of physical activity) to 4 (high adherence to the MD and 4 or more hours/week of physical activity).

### Neurocognitive outcomes

Cognitive impairment at the baseline was defined as a MMSE score one standard deviation (SD) below the population mean value [34]. Cognitive decline was defined as a change in MMSE score (unadjusted) to the worst 25% of the distribution of change for the whole sample as previously reported [22]. MMSE assessments were available for the Pro.V.A. (baseline, 4- and 7-year follow-up) and ILSA (baseline, 3- and 8-year follow-up) studies. Dementia variables were available at baseline and follow-up for the Pro.V.A. and ILSA studies, and at follow-up for the BEST-FU study, which included only dementia-free community-dwelling subjects. The ILSA dementia variable was based on clinical diagnosis: DSM III-R criteria for the dementia syndrome (American Psychiatric Association 1987), the NINCDS-ADRDA criteria for possible and probable Alzheimer’s disease (AD) [35], the International Classification of Diseases (ICD-10) criteria for vascular dementia and other dementing diseases [36]. For the Pro.V.A. study, hospital discharge records (ICD-9 codes) and causes of death (ICD-9 codes) were used to identify participants with dementia. For the BEST-FU study, an algorithm based on a deterministic record linkage between the baseline cohort and the regional registry of drug prescriptions (using Anatomical Therapeutic Classification—ATC), the hospital discharge registry (ICD-9/10), the mortality registry (ICD-9/10), and the drug reimbursement registry (exemption code) was used to identify the presence of dementia [27].

### Statistical analysis

Continuous variables were summarized as mean and standard deviation (SD) or median and interquartile range (IQR), while categorical variables were presented as counts and percentages. Normal distributions of continuous variables were tested using the Shapiro–Wilk test. Harmonized variables were compared using chi-squared or Fisher exact tests for categorical variables and generalized linear models after testing for homoscedasticity (Levene test) or Wilcoxon rank-sum test for continuous variables.

Pooled cohort data from the BEST-FU, ILSA, and Pro.V.A. studies were used to examine independent associations between MD adherence, MedEx, and risk of cognitive decline and dementia. Baseline characteristics of the pooled cohort were compared by MD adherence tertiles (first tertile of distribution (< 27 BEST; < 28 Pro.V.A.; < 29 ILSA), low adherence; second tertile (27–31 BEST; 28–32 Pro.V.A.; 29–33 ILSA), medium adherence; third tertile (≥ 31 BEST; ≥ 32 Pro.V.A.; ≥ 33 ILSA), high adherence), cognitive decline, and dementia incidence. Cox proportional hazards regression models were used to assess the association between the MD score (continuous or in adherence classes) or specific food groups and cognitive decline and dementia incidence during follow-up. When considering dementia as the outcome, we combined the medium and high SES categories due to the small number of participants with incident dementia in the high SES group (n = 13). Analyses were repeated considering MedEx variables. Models were adjusted for sex, age, marital status, SES, smoking habits, BMI, walking limitations, number of comorbidities (≥ 3), energy intake and study site. The proportional hazards assumption was tested using Schoenfeld residuals for the covariates.

Compound symmetry covariance structure and Tukey adjustment for multiple comparisons were considered. To test whether SES modified the association between MD and neurocognitive outcomes, multiplicative interactions between MD adherence and SES were evaluated, and analyses were then stratified by SES. In order to explore differences between the two sexes in the associations examined, sex-stratified analyses were also performed. All analyses were performed using the SAS (release 9.4 SAS Institute Inc., Cary, NC) statistical package. Two-tailed p-values < 0.05 were considered statistically significant.

## Results

The pooled cohort consists of 8,568 subjects, aged between 42 and 101 years (mean age 72.3 ± 9.6 years), 52.4% female. The characteristics of the cohort pooled and by original study are shown in Supplementary Table S2.

Baseline characteristics associated with MD score adherence are shown in Table 2. A higher MD adherence was more common in men, participants with higher education and SES, higher median daily energy intake, former smokers, and those with higher MMSE scores at baseline.

**Table 2:** Baseline characteristics, by MD score adherence

	1st tertile° (n = 2,776)	2nd tertile° (n = 2,997)	3rd tertile° (n = 2,795)	p-value
*Socio-demographic variables*				
Age, mean ± SD	72.2 ± 9.7	72.1 ± 9.7	72.6 ± 9.3	0.0843
Sex, n (%)				< 0.0001
Women Men	1567 (56.5) 1209 (43.5)	1548 (51.7) 1449 (48.3)	1375 (49.2) 1420 (50.8)	
Education, n (%)				< 0.0001
Primary school or less Middle school High school University or higher	2163 (78.4) 353 (12.8) 162 (5.9) 81 (2.9)	2258 (75.6) 423 (14.2) 215 (7.2) 91 (3.1)	2009 (72.1) 375 (13.5) 262 (9.4) 141 (5.1)	
Work done for most of the time, n (%)				< 0.0001
Housewife Blue collar White collar	485 (17.9) 1542 (57.0) 680 (25.1)	285 (13.2) 1627 (55.6) 914 (31.2)	373 (13.7) 1434 (52.5) 924 (33.8)	
Marital status, n (%)				0.0066
Single or never married Married or cohabiting Separated or divorced Widowed	174 (6.3) 1616 (58.3) 34 (1.2) 950 (34.3)	207 (6.9) 1862 (62.2) 23 (0.8) 901 (30.1)	182 (6.5) 1732 (62.0) 27 (1.0) 853 (30.5)	
SES^§^, n (%)				< 0.0001
1 low 2 medium 3 high	2053 (74.0) 521 (18.8) 201 (7.2)	2039 (68.1) 690 (23.1) 265 (8.9)	1825 (65.3) 613 (21.9) 357 (12.8)	
*Nutritional status*				
BMI, kg/m^2^, mean ± SD	27.2 ± 4.6	27.2 ± 4.5	27.0 ± 4.2	0.1768
BMI, n (%)				0.3461
< 18.5 kg/m^2^ 18.5–24.9 kg/m^2^ 25–29.9 kg/m^2^ ≥ 30 kg/m^2^	33 (1.5) 690 (30.3) 1000 (43.9) 554 (24.3)	34 (1.3) 804 (31.0) 1160 (44.7) 598 (23.0)	30 (1.2) 769 (31.9) 1098 (45.5) 514 (21.3)	
Energy intake, kcal, median (Q1, Q3)	2810 (2519, 3282)	2816 (2582, 3257)	2824 (2537, 3286)	0.2111
*Lifestyle and health status variables*				
Smoking status, n (%)				< 0.0001
Current smoker Former smoker Never smoker	543 (19.6) 755 (27.3) 1473 (53.2)	520 (17.4) 972 (32.5) 1499 (50.1)	484 (17.3) 945 (33.8) 1365 (48.9)	
Alcohol consumption, n (%)				< 0.0001
Heavy consumer* Light consumer** No consumer	408 (14.7) 531 (19.2) 1833 (66.1)	519 (17.3) 1101 (36.8) 1376 (45.9)	599 (21.4) 1306 (46.7) 889 (31.8)	
Number of comorbidities ≥ 3, n (%)	1727 (62.2)	1861 (62.1)	1689 (60.5)	0.3100
Number of medications ≥ 5, n (%)	499 (18.8)	485 (16.9)	422 (16.0)	0.0243
Limited mobility, cannot walk, n (%)	145 (7.7)	92 (4.4)	87 (4.3)	< 0.0001
Physical activity, ≥ 4 h/week, n (%)	176 (14.1)	312 (19.2)	271 (19.1)	0.0005
MMSE at T0, mean ± SD	24.6 ± 4.9	24.9 ± 4.6	25.2 ± 4.4	< 0.0001

°1st tertile of distribution (< 27 BEST; < 28 Pro.V.A.; < 29 ILSA), low adherence; 2nd tertile (27–31 BEST; 28–32 Pro.V.A.; 29–33 ILSA), medium adherence; 3rd tertile (≥ 31 BEST; ≥ 32 Pro.V.A.; ≥ 33 ILSA), high adherence)

Abbreviations: MD, Mediterranean Diet; MMSE, Mini-Mental State Examination; Q1, Quartile 1; Q3, Quartile 3; SD, Standard Deviation; SES, Socio-Economic Status

^§^ SES composite measure was defined considering education (primary school or middle school: score 1; high school or university or more: score 2) and work done for most of time (blue collar or housewife: score 1; white collar: score 2). Total score 1,2 corresponded to SES = 1 (low); total score 3 to SES = 2 (medium); total score 4 to SES = 3 (high)

^*^ Heavy consumer ≥ 7 AU/week women; ≥ 14 AU/week men

^**^ Light consumer < 7 AU/week women; < 14 AU/week men

**Fig. 2:**
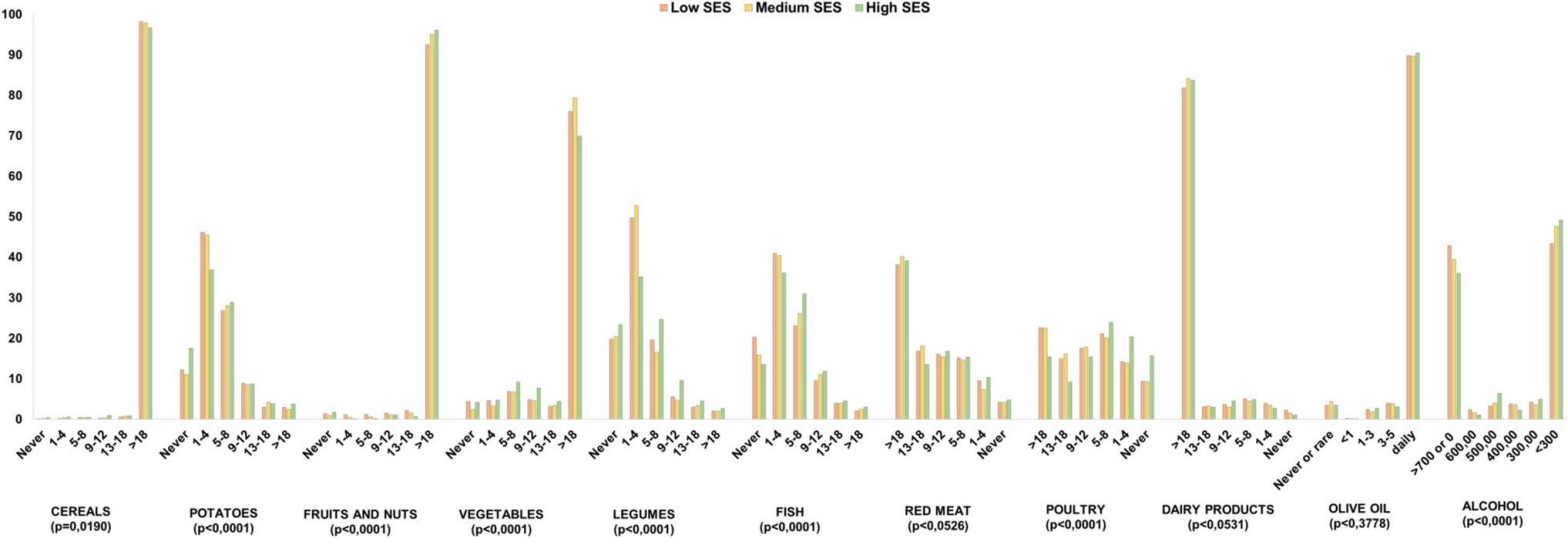
Distribution of consumption of food groups according to SES

Figure 2 shows a comparison of food group intakes (portions per month) by SES level. We observed that people with higher economic status were more likely to consume potatoes, fruit and nuts, legumes and fish, and less likely to consume cereals, red meat and products, poultry, dairy products and alcoholic beverages.

Cognitive decline on the MMSE was observed during follow-up (median 8 years) in 1630 participants (38.1% with available data) and was not associated with MD adherence.

**Fig. 3:**
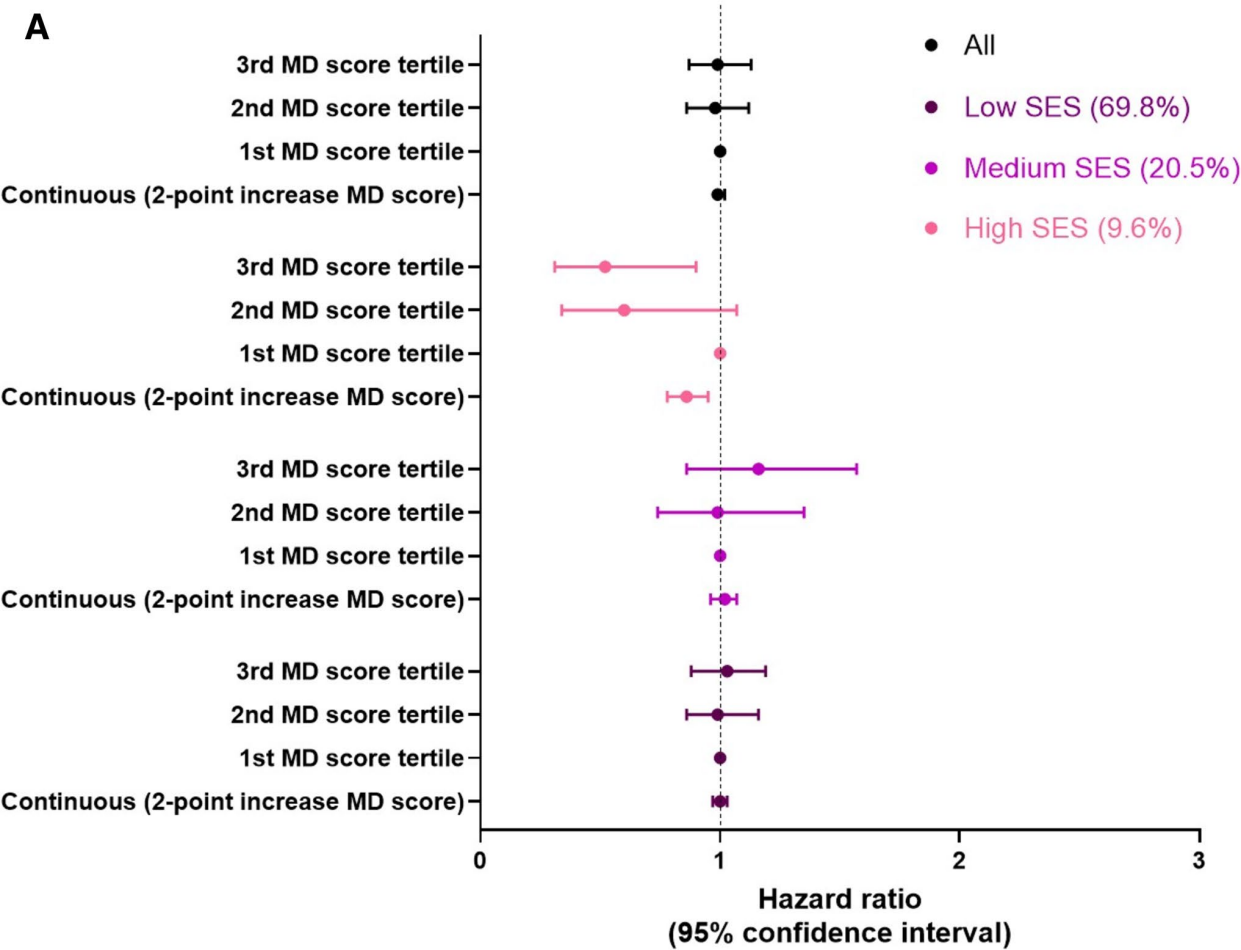

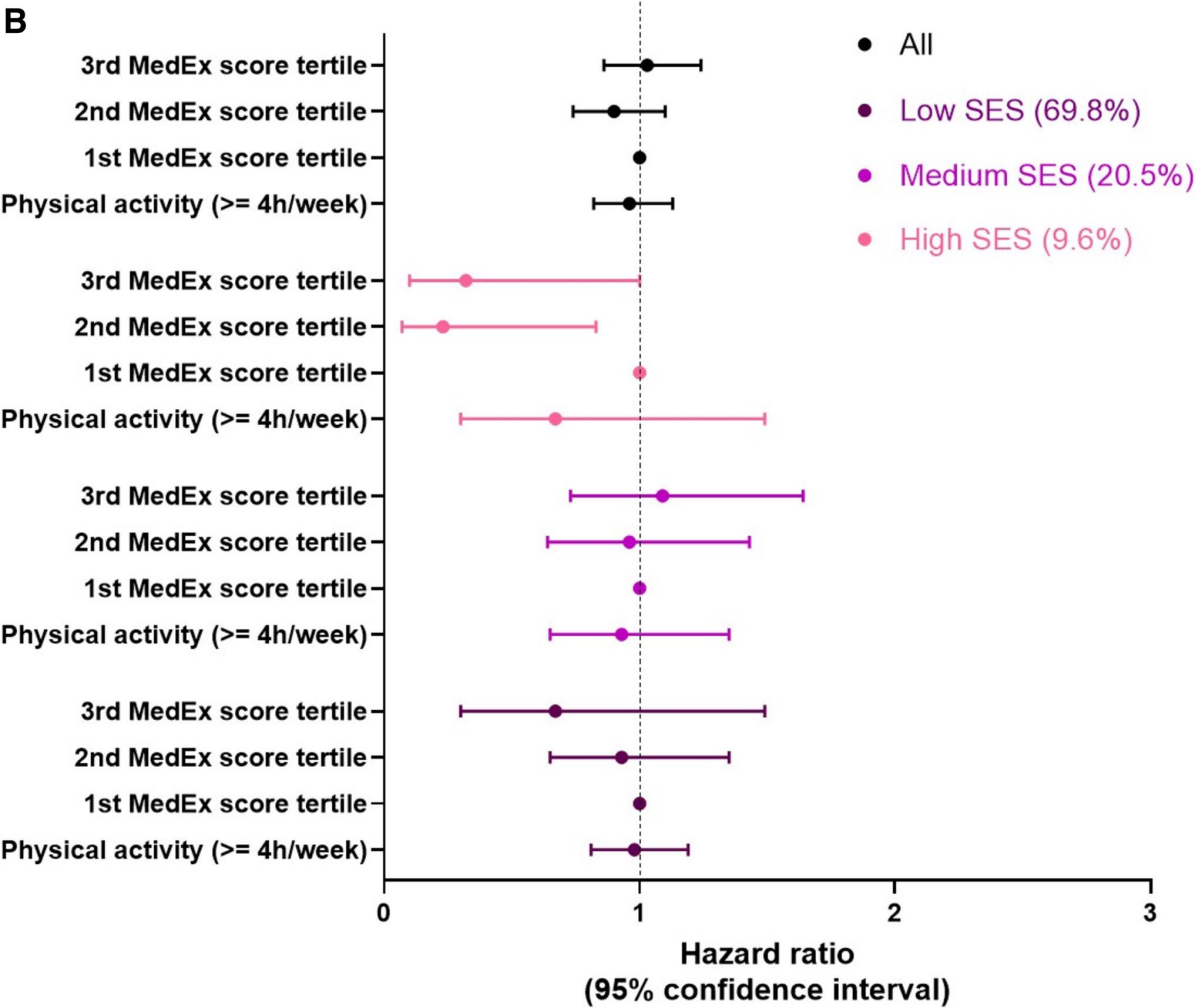
**A** Adjusted hazard ratios (HR) (95% CI) of cognitive decline for MD. Notes: Model adjusted for age, sex, marital status, SES, BMI, energy intake, smoking status, walking limitations, number of comorbidities ≥ 3, energy intake, study site. Abbreviations: MD, Mediterranean Diet; HR, Hazard Ratio; CI, Confidence Intervals. **B** Adjusted hazard ratios (HR) (95% CI) of cognitive decline for MedEx. Notes: Model adjusted for age, sex, marital status, SES, BMI, energy intake, smoking status, walking limitations, number of comorbidities ≥ 3, energy intake, study site. Abbreviations: MD, Mediterranean Diet; HR, Hazard Ratio; CI, Confidence Intervals

In fully adjusted Cox proportional hazards models (Table 3), the MD adherence score was not significantly associated with cognitive decline, either considering the continuous or categorized score, and similar results were observed for the MedEx score. We found a statistically significant interaction between MD and SES (p for interaction = 0.02) but not between MedEx and SES (p for interaction = 0.11) when considering cognitive decline as an outcome. When the analysis was stratified by SES, only in the high SES group, each 2-point increase in MD adherence was associated with a 14% reduction in the risk of cognitive decline. Furthermore, high adherence to the MD corresponded to a 48% lower risk of cognitive decline (HR 0.52, 95%CI 0.31–0.90) (Fig. 3A). Among participants in the high SES group, the second and third tertiles of the MedEx score were associated with a 77% (HR 0.23, 95%CI 0.07–0.83) and 68% (HR 0.32, 95%CI 0.10–1.00) reduction in the risk of cognitive decline, respectively, compared with the first tertile (Fig. 3B).

**Table 3:** Hazard ratios for the association of MD adherence score and MedEx with cognitive decline on the MMSE*

	Hazard Ratio (95% Confidence Interval)			
	Overall cohort**	SES = 1 (low)	SES = 2 (medium)	SES = 3 (high)
MD score continuous, 2-point increase	0.99 (0.97–1.02)	1.00 (0.97–1.03)	1.02 (0.96–1.07)	0.86 (0.78–0.95)
MD score, categorized				
1st tertile 2nd tertile 3rd tertile	1.00 0.98 (0.86–1.12) 0.99 (0.87–1.13)	1.00 0.99 (0.86–1.16) 1.03 (0.88–1.19)	1.00 0.99 (0.74–1.35) 1.16 (0.86–1.57)	1.00 0.60 (0.34–1.07) 0.52 (0.31–0.90)
Physical activity, ≥ 4 h/week	0.96 (0.82–1.13)	0.98 (0.81–1.19)	0.93 (0.65–1.35)	0.67 (0.30–1.49)
MedEx^§^, 1-point increase	1.01 (0.94–1.09)	1.02 (0.93–1.12)	1.02 (0.86–1.22)	0.83 (0.54–1.27)
MedEx^§^				
1st tertile 2nd tertile 3rd tertile	1.00 0.90 (0.74–1.10) 1.03 (0.86–1.24)	1.00 0.90 (0.72–1.12) 1.04 (0.84–1.29)	1.00 0.96 (0.64–1.43) 1.09 (0.73–1.64)	1.00 0.23 (0.07–0.83) 0.32 (0.10–1.00)

Abbreviations: MD, Mediterranean Diet; HR, Hazard Ratio; CI, Confidence Interval

^§^: available only for the Pro.V.A. study

^*^: only participants not demented at the baseline

**: model adjusted for age, sex, marital status, SES, BMI, energy intake, smoking status, walking limitations, number of comorbidities ≥ 3, energy intake, study site

Incident dementia was observed in 238 participants (4.2%, median follow-up 8 years). Adherence to the MD and MedEx scores was not significantly associated with incident dementia in fully adjusted Cox models (Table 4**)**, but PA alone was statistically significantly inversely associated with incident dementia (HR 0.38, 95%CI 0.16–0.91). No statistically significant interactions were found when considering the interaction between SES and MedEx on incident dementia (MD*SES p = 0.16; MedEx*SES p = 0.89). Sex-stratified analyses showed no differences between the two sexes in the associations examined (data not shown).

**Table 4:** Hazard ratios for the association of MD adherence score and MedEx with incidence of dementia*

	Hazard Ratio (95% Confidence Interval)		
	Overall cohort**	SES = 1 (low)	SES = 2 (medium or high)
Continuous, 2-point increase	0.93 (0.87–1.00)	0.91 (0.83–1.00)	0.97 (0.84–1.12)
Classes,			
1st tertile 2nd tertile 3rd tertile	1.00 0.81 (0.54–1.23) 0.76 (0.50–1.15)	1.00 0.65 (0.40–1.07) 0.67 (0.41–1.09)	1.00 1.38 (0.61–3.13) 1.15 (0.49–2.69)
Physical activity, ≥ 4 h/week	0.38 (0.16–0.91)	0.38 (0.13–1.08)	0.97 (0.76–1.23)
MedEx^§^, 1-point increase	0.77 (0.57–1.03)	0.81 (0.56–1.16)	0.73 (0.42–1.26)
MedEx^§^			
1st tertile 2nd tertile 3rd tertile	1.00 0.77 (0.40–1.48) 0.64 (0.33–1.24)	1.00 0.46 (0.19–1.08) 0.70 (0.33–1.51)	1.00 1.17 (0.37–3.76) 0.52 (0.14–2.01)

Abbreviations: MD, Mediterranean Diet; HR, Hazard Ratio; CI, Confidence Intervals

^§^: available only for the Pro.V.A. and BEST-FU studies

^*^: only participants not demented at the baseline

**: model adjusted for age, sex, marital status, SES, BMI, energy intake, smoking status, walking limitations, number of comorbidities ≥ 3, energy intake, study site

## Discussion

In this large, pooled sample of Italian community-dwelling adults, we examined the effects of MD alone and in combination with PA on cognitive decline and dementia incidence over a median follow-up of 8 years. We found that around one out of three individuals had a high adherence to MD, and that the prevalence of high SES was higher among participants with high MD adherence compared to those with low MD adherence (12.8% vs 7.2%). This is consistent with previous studies showing the key role of SES in promoting adherence to MD and healthy eating guidelines [17, 37, 38]. Furthermore, when looking at individual food groups, we also observed that people with higher economic status were more likely to consume cereals, fruits, nuts, seeds, vegetables, and fish, confirming data from a large body of epidemiological data showing that diet quality follows a socioeconomic gradient [11, 17]. The higher costs associated with a healthy diet may partly explain why low MD adherence is more common among the more deprived populations [11, 39].

We also found no evidence of an association between MD adherence and cognitive decline and dementia incidence in the total sample. However, when we stratified the analysis by SES, we observed that only in the high SES group, people with higher adherence to MD had a reduced risk of cognitive decline. To our knowledge, this is the first study to show that the association of MD—alone and in combination with physical exercise—with cognitive risk differs by SES group in the Italian population. Indeed, few studies investigated SES as an effect modifier of the relationship between dietary habits and cognitive function, and their results remain inconsistent. Similar to our data, in the ELSA-Brazil study, adherence to the MIND diet was not associated with cognition overall. However, stratified by income, higher adherence related to better executive function in high-income individuals, but to poorer global cognition and executive function in low-income groups [17]. In the same cohort, higher adherence to the planetary health diet was found to be associated with slower memory decline and global cognition in high-income participants only [40]. Similarly, Parrot et al. found that a prudent dietary pattern was linked to better cognitive performance only among older Canadians with higher SES [18]. Additionally, Ou and colleagues observed that the dementia-reducing benefits of a healthy lifestyle were most pronounced in high-SES participants from the UK Biobank cohort [41]. In contrast, Koyama et al. reported that higher adherence to the MD slowed cognitive decline in black, but not white, older Americans, suggesting that socioeconomic factors may underlie ethnic differences in dementia incidence [42]. Moreover, two other studies noted significant interactions between SES and lifestyle scores, with some findings indicating that financially disadvantaged individuals might benefit more cognitively from healthy lifestyles [19, 20].

Several considerations need to be made to explain our findings. Individuals with higher SES may benefit from better access to high-quality fresh foods that are often rich in vitamins and minerals which contribute to brain health. These foods have a low energy density—such as fruits, vegetables, whole grains, and fish—that are essential components of the MD [11, 39], which enhances diet adherence and its associated cognitive benefits. Furthermore, higher SES is associated with greater educational opportunities and improved health literacy, facilitating the acquisition of nutritional knowledge and understanding of medical recommendations. This not only contributes to greater adherence to clinical recommendations but also to the ability to make informed health decisions and encourage more informed preventive behavior [10, 11, 38] [43–45]. This could make public health nutrition messaging more cost-effective for high-SES groups [41]. Furthermore, the enhanced economic resources and access to healthcare in these communities facilitate earlier diagnosis of cognitive impairment, due to the possibility of regular check-ups and dedicated screening programs. This favorable environment allows prompt identification of early symptoms, which could impact the progression of the condition [46]. Moreover, higher education and/or occupational complexity understood as constant mental engagement and exposure to cognitively stimulating tasks, also contribute to increased cognitive reserve, delaying the onset of dementia [12, 13]. Finally, lower levels of chronic stress and economic hardship in high-SES individuals further support brain health and cognitive function [20, 47]. From a biological perspective, MD may enhance cognitive function by providing abundant antioxidants (e.g., vitamins C and E, carotenoids, polyphenols) and a healthy fatty acid profile characterized by low saturated/trans fats and high mono- and polyunsaturated fats (including long-chain omega-3 fatty acids). These components likely mitigate oxidative stress, beta-amyloid deposition, and inflammation, while promoting neurogenesis and improving synaptic membrane fluidity [48, 49]. B vitamins also support brain function via energy production, nucleic acid repair, methylation, and neurochemical synthesis [50]. Additionally, MD may favorably modulate gut microbiota by reducing dysbiosis, a factor implicated in cognitive decline and Alzheimer’s disease [23].

Moreover, the MD pattern’s benefits may extend to socio-cultural aspects—such as eating locally produced and seasonal ingredients and sharing convivial meals [51]—which is particularly important for reducing the risk of cognitive deficits, given the well-documented links between increased social interactions and reduced risk of neurodegeneration [52]. Additionally, social connectedness should be considered alongside dementia and higher SES, as individuals with higher SES are often less socially isolated and tend to have stronger social and support networks, which may play a key role in maintaining cognitive function [53].

When looking at PA, we only found a trend in the association between PA and cognitive decline, especially in the high SES group, but this was not statistically significant. This may be due to the small sample size, as this variable was only available for the Pro.V.A. study. The association only became significant when physical activity was combined with MD in MedEx.

Considering incident dementia as the outcome, we found no significant association with MD and MedEx, even when the sample was stratified by SES. This finding contrasts with previous studies reporting a protective role of MD on all-cause dementia [3, 54]. A possible explanation for this discrepancy may be the short follow-up period (8 years), which may have reduced our ability to detect long-term effects of diet on cognitive change, as previously suggested by Féart and colleagues [55]. Another possible explanation is that the overall incidence of dementia, which is 4.2%, is lower than the prevalence of dementia reported in Western countries, that is around 8% in people over 65 years of age, and rises to over 20% after the age of 80 [56]. This is mainly due to the fact that in both Pro.V.A. and BEST-FU, the diagnosis of incident dementia was based on record linkage with health registers. Although previous research has demonstrated that routinely collected health data with record linkage can provide plausible estimates of dementia prevalence and incidence, underdiagnosis may still occur, potentially leading to underestimation of associations with risk/protective factors [57]. Instead, in line with the current literature [5, 6], we found that PA reduced the risk of dementia by 62% in the whole sample, irrespective of SES. Aerobic and muscle-strengthening exercises have been shown to prevent and delay dementia, possibly by increasing brain-derived neurotrophic factor, which protects against hippocampal atrophy and helps preserve cognitive function [58]. Furthermore, structural and environmental factors, such as access to green spaces, low crime rates, and walkable neighborhoods, significantly influence PA levels, particularly outdoor exercise.

### Strengths and limitations

The present paper has several strengths. The three studies have similar characteristics in terms of the geographical area from which participants were recruited, the method of data collection, and the community-based setting, which facilitates comparison between the studies. They have a prospective design so that exposures were assessed before the development of outcomes, which limits recall and selection bias and reverse causation. In addition, we attempted to standardize the harmonization of exposures, outcomes, and confounders to remove potential sources of heterogeneity and non-comparability. We examined a number of potential confounders, which allowed us to control for their potential confounding effects in the analysis. In addition, adherence to MD was assessed using an a priori score that is consistent with the principles of the Mediterranean diet and is not specific to the dietary intake of the population studied. The advantages of the MD include the weighting of the selected food groups based on the frequency of consumption (thresholds were chosen according to an a priori hypothesis) and independent of the consumption of the sample studied [59].

However, the study has important limitations because it was designed retrospectively, so there is heterogeneity in how the different studies were designed and conducted, including study objectives, sampling frames, recruitment procedures, and data collection. In particular, the ILSA study lacked a complete baseline assessment of physical activity levels; to overcome this limitation, a proxy for mobility was used by considering the ability to walk, which was also available for the Pro.V.A. Additionally, we cannot exclude the possibility of differential control for confounders between them, as non-dietary variables varied between studies. However, to minimize the between-studies heterogeneity on the confounding variables, we standardized the confounders selected for inclusion in the statistical models and their categorization. Furthermore, dietary intake was self-reported, so inaccuracies in dietary recall may have affected the observed association. Moreover, the dietary score does not assess the quality of the food consumed within the same group. For example, the high-income segment may consume the same amount of extra virgin olive oil (EVOO), but we know that fresh, high-quality EVOO, which contains higher levels of polyphenols and vitamins, costs more than the low-quality EVOO found in stores. The same could be true for certain vegetables, such as iceberg lettuce versus spinach. In addition, more disadvantaged individuals may use the same ingredients in less healthy recipes due to their likely lower nutritional literacy. In addition, data on possible dietary changes thereafter were not available, although we expect dietary patterns to remain fairly stable over time, especially in older adults [60]. Additionally, older cohorts are particularly susceptible to loss to follow-up. However, if participants who dropped out were more likely to have a poorer diet and more cognitive decline than those who remained in the study, the results would most likely be biased towards the null [16]. Finally, although we controlled for all potential known confounders, we cannot exclude the possibility of residual confounding of our results on MD and cognitive outcomes (i.e. apolipoprotein E polymorphism, the main genetic determinant of AD) [61].

## Conclusion

In conclusion, our study suggests that adherence to the MD is associated with cognitive decline according to socioeconomic level. Specifically, greater adherence to the MD was associated with better cognitive performance over time only in participants with high SES. This is the first large population-based cohort study to reveal socioeconomic inequalities in the cognitive protection provided by MD and PA among older Italian adults. Future longitudinal studies using a life course approach are needed to investigate these associations across different socioeconomic settings and to explore the underlying mechanisms. From a public health perspective, these findings highlight the need for more effective strategies to reduce socioeconomic disparities. Such strategies should not only promote healthy dietary habits but also ensure equitable access to nutritious foods, essential steps in preventing cognitive deterioration, enhancing overall health, and lowering healthcare costs. However, addressing dietary and lifestyle interventions within the context of systemic inequities remains challenging. Modern public health views these issues as structural rather than individual failings, emphasizing approaches that respect the lived experiences of affected populations. Thus, the responsibility for unhealthy behaviors must extend beyond individual choices to include broader public health interventions. Targeted health campaigns that reduce the risk of cognitive decline by considering complex social determinants are particularly important.

## Supplementary Information

Below is the link to the electronic supplementary material.

Supplementary file1 (DOCX 27 KB)

## Data Availability

No datasets were generated or analysed during the current study.

## Abbreviations

SES: Socioeconomic status
MD: Mediterranean diet
PA: Physical activity
PROMED-COG: PROtein enriched MEDiterranean diet to combat undernutrition and promote healthy neuroCOGnitive ageing in older adults
ILSA: Italian Longitudinal Study of Aging
Pro.V.A.: Progetto Veneto Anziani study
BEST-FU: Bollate Eye Study Follow up
MMSE: Mini-mental state examination
BMI: Body mass index
FFQ: Food Frequency Questionnaire
AD: Alzheimer’s disease
ICD: International Classification of Diseases
SD: Standard deviation
IQR: Interquartile ranges
HR: Hazard ratio
CI: Confidence interval
