# Supplementary material for: Socioeconomic status modifies the association between adherence to the Mediterranean diet and cognitive outcomes: results from the Collaborative PROMED-COG Pooled Cohorts Study
Source: Soc Psychiatry Psychiatr Epidemiol. 2025 Sep 29;61(4):663–77. doi: 10.1007/s00127-025-02993-2 (PMC13021694; doi:10.1007/s00127-025-02993-2)
Supplement: Supplementary file 1 — Supplementary file1 (DOCX 27 KB) [file 127_2025_2993_MOESM1_ESM.docx]

**Supplementary Table S1. Individual foods and common food groups included in the calculation of the MD score.**

|  | **BEST-FU** | **ILSA** | **PRO.V.A.** |
| --- | --- | --- | --- |
| **Fruits** | Apricot, orange, banana, cherry, watermelon, fig, lemon, strawberry, clementines and tangerines, pear, apple, peach, kiwi, plum, grape, fruit juice | Fresh fruits (orange, apple, banana, pear, peach, etc), dried fruits (nut, almond, etc), fruit juice | Raw fruits, cooked fruits (generic), fruit homogenate, orange juice |
| **Vegetables** | Green salad, tomatoes, fennel raw and cooked, celery, asparagus, cucumber, carrots raw and cooked, zucchini, pepper, swiss chard, artichoke, cauliflower and broccoli, onion, mushrooms, aubergine, spinach, cabbage, mixed vegetables, green beans, tomato sauce, tomato paste | Fresh vegetables | Green salad or mixed vegetables (generic), tomato sauce |
| **Potatoes** | Potatoes and pureè, soft pasta with potatoes | Potatoes | Potatoes |
| **Cereals** | Bread, dry pasta, rice, macaroni, egg pasta, semolina, pasta prepared with broth, pasta and beans, polenta, orecchiette, rice salad | Bread, pasta, rice | Wholemeal bread, polenta, pasta (alone or prepared with broth), rice, semolina |
| **Legumes** | Beans, peas | Fresh legumes, dried legumes | Legumes (generic), minestrone (with potatoes and legumes) |
| **Fish and sea products** | Dover sole, mackerel, anchovies, trout, hake, eel | Sole, trout, cod, bream, snapper, mackerel, fresh tuna, bluefish, salmon | Fish (generic) |
| **Red meat and products** | Beef canned, beef lean and fatty cuts, horse, cutlet, pork chop, stew with potatoes or peas,  stew beef, meet with tuna sauce, lamb, offals, , cured ham, sausages, coppa, bacon, ham, bresaola, speck, salami | Beef, horse, pork, game, beef canned, sausages, ham, salami, mortadella, organ meats | Ham, prosciutto/speck, salami/mortadella/soppressa, meat homogenate (generic), meat sauce |
| **Poultry** | Turkey, rabbitt, chicken leg and breasts, game bird | Turkey, rabbitt, chicken | White meat (generic), meat homogenate (generic) |
| **Full-fats dairy products** | Whole milk, low-fat milk, skimmed milk, whole yogurt, light yogurt processed cheese, crescenza, parmesan, pecorino, ricotta, emmental, mozzarella, provola sweet and hot, mascarpone, milk ice cream, fruit ice cream | Whole milk, whole yogurt, mascarpone, gorgonzola, processed cheese, parmesan, emmenthal, mozzarella, ricotta, butter | Cottage/fresh cheese (e.g. ricotta, mozzarella, stracchino), mature cheese (asiago, parmesan, emmenthal, brie, taleggio, formaggini, mascarpone, cheese slices), grated parmesan, cooking cream, butter, margarine, cappuccino, milk, yogurt |
| **Alcoholic**  **beverages** | Beer, red wine, rose wine, bitters, dessert wine, whiskey | Spirits, dessert wine, cherry, beer, wine (red, white and rose) | Spirits, dessert wine, cherry, beer, wine, alcohol |
| **Olive oil** | Olive oil | Olive oil | Olive oil |

**Supplementary Table S2. Baseline characteristics, by study**

|  | **Overall cohort**  **(n=8,568)** | **BEST-FU**  **(n=1,353)** | **Pro.V.A.**  **(n=2,981)** | **ILSA**  **(n=4,234)** | **p-value** |
| --- | --- | --- | --- | --- | --- |
| *Socio-demographic variables* |  |  |  |  |  |
| Age, mean±SD | 72.3±9.6 | 56.8±8.1 | 76.0±7.7 | 74.6±5.6 | <0.0001 |
| Sex, n (%)  Women  Men | 4490 (52.4)  4078 (47.6) | 681 (50.3)  672 (49.7) | 1762 (59.1)  1219 (40.9) | 2047 (48.4)  2187 (51.7) | <0.0001 |
| Education, n (%)  Primary school or less  Middle school  High school  University or higher | 6430 (75.4)  1151 (13.5)  639 (7.5)  313 (3.7) | 776 (57.4)  420 (31.0)  131 (9.7)  26 (1.9) | 2615 (88.0)  212 (7.1)  96 (3.2)  49 (1.7) | 3039 (72.2)  519 (12.3)  412 (9.8)  238 (5.7) | <0.0001 |
| Work done for most of the time, n (%)  Housewife  Blue collar  White collar | 1243 (14.9)  4603 (55.0)  2518 (30.1) | 98 (7.2)  722 (53.4)  533 (39.4) | 357 (12.1)  1794 (60.9)  794 (27.0) | 788 (19.4)  2087 (51.3)  1191 (29.3) | <0.0001 |
| Marital status, n (%)  Single or never married  Married or cohabiting  Separated or divorced  Widowed | 563 (6.6)  5210 (60.9)  84 (1.0)  2704 (31.6) | 47 (3.5)  1151 (85.0)  23 (1.7)  132 (9.8) | 227 (7.6)  1536 (51.5)  16 (0.5)  1201 (40.3) | 289 (6.8)  2523 (59.7)  45 (1.1)  1371 (32.4) | <0.0001 |
| SES^§^, n (%)  1 low  2 medium  3 high | 5917 (69.1)  1824 (21.3)  823 (9.6) | 816 (60.3)  384 (28.4)  153 (11.3) | 2171 (72.9)  679 (22.8)  130 (4.4) | 2930 (69.3)  761 (18.0)  540 (12.8) | <0.0001 |
| *Nutritional status* |  |  |  |  |  |
| BMI, kg/m^2^, mean±SD | 27.2±4.4 | 27.0±4.1 | 27.6±4.6 | 26.1±3.6 | <0.0001 |
| BMI, n (%)  <18.5 kg/m^2^  18.5-24.9 kg/m^2^  25-29.9 kg/m^2^  ≥30 kg/m^2^ | 97 (1.3)  2263 (31.1)  3258 (44.7)  1666 (22.9) | 9 (0.7)  439 (32.7)  612 (45.6)  281 (21.0) | 40 (1.4)  799 (28.0)  1278 (44.6)  742 (26.0) | 48 (1.6)  1025 (33.2)  1368 (44.4)  643 (20.9) | <0.0001 |
| Energy intake, kcal, median (Q1, Q3) | 2813.8  (2537, 3276) | 2841  (2194, 3586) | 2686  (2652, 2863) | 2986  (2258, 3503) | <0.0001 |
| MD score (Panagiotakos), n (%)  1^st^ tertile  2^nd^ tertile  3^rd^ tertile | 2776 (32.4)  2997 (35.0)  2795 (32.6) | 455 (33.6)  505 (37.3)  393 (29.1) | 800 (26.8)  1134 (38.1)  1047 (35.1) | 1521 (35.9)  1358 (32.1)  1355 (32.0) | <0.0001 |
| *Lifestyle and Health status variables* |  |  |  |  |  |
| Smoking status, n (%)  Current smoker  Former smoker  Never smoker | 1547 (18.1)  2672 (31.2)  4337 (50.7) | 698 (51.6)  305 (22.5)  350 (25.9) | 264 (8.9)  892 (29.9)  1824 (61.2) | 585 (13.8)  1475 (34.9)  2163 (51.2) | <0.0001 |
| Alcohol consumption, n (%)  Heavy consumer*  Light consumer**  No consumer | 1526 (17.8)  2938 (34.3)  4098 (47.9) | 228 (16.8)  821 (60.7)  304 (22.5) | 364 (12.2)  531 (17.8)  2086 (70.0) | 934 (22.1)  1586 (37.5)  1708 (40.4) | <0.0001 |
| Number of comorbidities ≥3, n (%) | 5277 (61.6) | 448 (33.1) | 2028 (68.0) | 2801 (66.2) | <0.0001 |
| Number of medications ≥5, n (%) | 1406 (17.2) | 178 (13.2) | 644 (26.2) | 593 (14.5) | <0.0001 |
| Mobility limitations, cannot walk, n (%) | 324 (5.4) | na | 243 (8.2) | 81 (2.7) | <0.0001 |
| Physical activity ≥4 h/week, n (%) | 759 (17.7) | 70 (5.4) | 689 (23.1) | na | <0.0001 |
| MedEx, n (%)  1^st^ tertile  2^nd^ tertile  3^rd^ tertile | 1069 (24.9)  1487 (34.7)  1733 (40.4) | 420 (32.1)  488 (37.3)  400 (30.6) | 649 (21.8)  999 (33.5)  1333 (44.7) | na | <0.0001 |
| MMSE at T0, mean±SD | 24.9±4.6 | na | 23.7±5.2 | 26.1±3.6 | <0.0001 |
| Dementia at T0 | 280 (3.6) | 0 (0.0) | 21 (0.7) | 259 (7.6) | <0.0001 |

^§^: SES composite measure defined considering education (primary school or middle school: score 1; high school or university or more: score 2) and work done for most of time (blue collar or housewife: score 1; white collar: score 2). Total score 1,2 corresponded to SES=1 (low); total score 3 to SES=2 (medium); total score 4 to SES=3 (high)

*: ≥7 AU/week women; ≥14 AU/week men; **: <7 AU/week women; <14 AU/week men

Abbreviations: MD, Mediterranean diet; MMSE, Mini-Mental State Examination; na, not available Q1, Quartile 1; Q3, Quartile 3; SD, Standard Deviation; SES, Socio-Economic Status.
